# Longitudinal volume analysis from computed tomography: Reproducibility using adrenal glands as surrogate tumors

**DOI:** 10.4103/0971-6203.62130

**Published:** 2010

**Authors:** Nicolas D. Prionas, Marijo A. Gillen, John M. Boone

**Affiliations:** Department of Radiology, University of California Davis Medical Center, Ellison Ambulatory Care Center, 4860 Y Street Suite 3100, Sacramento, CA, USA

**Keywords:** Computed tomography, volumetric, reproducibility

## Abstract

This study aims to determine the precision (reproducibility) of volume assessment in routine clinical computed tomography (CT) using adrenal glands as surrogate tumors. Seven patients at our institution were identified retrospectively as having received numerous abdominal CT scans (average 13.1, range 5 to 20). The adrenal glands were used as surrogate tumors, assuming no actual volume change. Left and right adrenal gland volumes were assessed by hand segmentation for each patient scan. Over 1240 regions of interest were outlined in total. The reproducibility, expressed as the coefficient of variation (COV), was used to characterize measurement precision. The average volumes were 5.9 and 4.5 cm^3^ for the left and right adrenal gland, respectively, with COVs of 17.8% and 18.9%, respectively. Using one patient’s data (20 scans) as an example surrogate for a spherical tumor, it was calculated that a 13% change in volume (4.2% change in diameter) could be determined with statistical significance at *P*=0.05. For this case, cursor positioning error in linear measurement of object size, by even 1 pixel on the CT image, corresponded to a significant change in volume (*P*=0.05). The precision of volume determination was dependent on total volume. Precision improved with increasing object size (r^2^ =0.367). Given the small dimensions of the adrenal glands, the ~18% COV is likely to be a high estimate compared to larger tumors. Modern CT scanners working with thinner sections (i.e. <1 mm) are likely to produce better measurement precision. The use of volume measurement to quantify changing tumor size is supported as a more precise metric than linear measurement.

## Introduction

Tumor volume change after therapy is thought to be predictive of therapeutic response for some cancers and cancer treatments.[1–3] The assessment of tumor response to a therapeutic regimen is often performed by measuring tumor size using computed tomography[4] (CT) or other modalities.[5–7] However, the precision of repeated volume measurements in CT using typical clinical scanning protocols is not well understood. The volume of an anatomical object in a patient assessed using CT can change as a result of a number of factors, including the alignment of the tumor (or object) relative to the CT sections, patient motion, observer outlining skill, the hardware and software tools used to delineate (mark) object boundaries, and the ability to clearly see the edge of the object. A tumor can also experience a true reduction in volume over time as a response to therapy, or it can increase in volume, generally indicating a lack of response. The clinical imaging protocol can also influence the accuracy of volume assessment. Parameters such as helical pitch, scan time, section thickness, the use of contrast agents, window/level settings, and patient positioning in the field of view are likely to have an effect on volume determination. Previous studies have explored the effects of various object and scan parameters on volumetric accuracy and precision using phantom models,[8,9] but few studies have evaluated these effects in real-world, clinical scenarios using computed tomography.[10]

When a measured change in tumor volume based on CT measurements does occur, how likely is this change due to actual tumor volume change as opposed to imprecision in the measurement? Previous studies by Vaidyanathan reported 17% inter-observer variability[11] in brain tumors imaged using MR. Gordon *et al* reported 12% to 13% measurement variability in pharyngeal cancer imaged on MR.[12] The purpose of this investigation was to explore the precision (reproducibility) of volume determination in routine clinical CT, exploiting a number of clinical cases at our institution where patients underwent numerous repeated abdominal CT scans over time as part of their clinical care. For repeated abdominal CT scans, a number of organs are visible and volumes can be determined. In some organs, barring large changes in weight of the patient, it would be likely that organ volume will not change over time or it will change very little.[13–15] With this in mind, the volumes of both adrenal glands were evaluated by boundary tracing, and the volume of each organ was assessed over time for each patient. Normal mean adrenal gland volumes have been reported to be 4.7 cm^3^ for women and 5.7 cm^3^ for men.[16] Although automatic segmentation routines are desirable to trace the outlines of a tumor or other anatomical structures, these routines are often unreliable and the manual outlining capabilities of a trained human observer remain the gold standard in this area. Previous studies have used human detection as the gold standard and have shown automatic and semi-automatic segmentation methods to have lower detection success rates and reduced similarity scores when compared to manual segmentation.[17,18] Thus, in this study, outlining the boundary of the object of interest was performed by a trained human observer.

This study focuses on *in vivo* measurements using typical CT protocols for abdominal imaging in a retrospectively evaluated patient population. The emphasis here, therefore, is assessment of real-world volume measurement reproducibility, as opposed to a situation where acquisition parameters have been optimized for quantitative volumetric accuracy (such as thin slice imaging).

## Materials and Methods

### Patient selection

Hospital records at the University of California Davis were used to identify a group of patients who had received a large number of serial abdominal CT scans. The sample population consisted of patients who were scanned as a result of trauma or other common clinical symptoms requiring abdominal CT. The imaging studies of seven patients were evaluated, under Institutional Review Board approval, which did not require patient consent (Protocol approval date: January 25, 2006). Only patients over the age of 18 were included in the study to ensure developmental growth did not influence organ volumetric measurements. The radiologist’s report accompanying each scan was consulted to assure that patients did not have remarkable disease in the organs of interest that would lead to changes in volume over time. CT scans that only partially imaged the organ of interest were not included. One patient (Patient 7) in the study had received a left adrenal gland resection; for this case unilateral evaluation was performed. The study’s patient population included four men and three women with ages ranging from 29 to 68 years old (mean, 50.6 years old). Other patient specific information is summarized in Table 1.

**Table 1:** Patient population information: Four men and three women were included in the study. Ages ranged from 29 to 68 years old. The number of abdominal CT scans received by a patient ranged from 5 to 20 scans

*Patient Number*	*Sex*	*Age (years)*	*# CT Scans*	*Time (years)*
1	M	29	16	1.50
2	F	49	12	3.42
3	M	68	15	4.50
4	M	44	5	0.83
5	F	68	20	3.17
6	M	55	6	0.42
7	F	41	18	5.25

All patient abdominal CT scans were performed on either a General Electric Lightspeed QX multi-slice scanner (Waukesha, WI, USA) or a General Electric CT/i single-slice scanner. Scanning parameters were determined by established clinical protocols at our institution. All scans were carried out at 120 kV with varied mAs. Section thickness was 5.0 mm, although in some instances CT section thickness varied from 5.0 to 10.0 mm. The majority of the 92 scans evaluated were contrast enhanced and 41 were specific to the examination of renal function.

### Analysis of patient images

**Figure 1:**
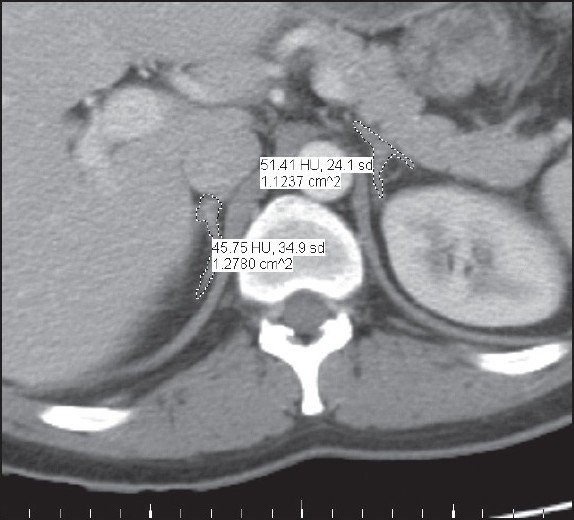
Adrenal Gland Segmentation: Hand-outlining of the regions of interest on one slice of a single scan for Patient 1. Over 160 adrenal volume measurements were made through the individual hand outlining of more than 1240 ROIs.

In total, 92 series of abdominal CT images were viewed using the picture archiving and communication system (PACS) at our institution and the built in image analysis software, iSite Enterprise version 3.5 (iSite Radiology, Philips, Eindhoven, Netherlands). Images were viewed and analyzed on a computer workstation by means of a 19-inch monitor (Tyco Electronics, Concord, CA, USA) with a 1280 × 1024 matrix. During organ boundary tracing (i.e. segmentation), images were magnified such that the organ of interest filled the entire screen. Typically, a minimum magnification of 400% (by area) was used and this magnification served to improve positioning fidelity of the pointing device, thereby reducing cursor positioning errors. All images were viewed consistently with the default abdominal window and level settings (window=400 HU, level=40 HU). All regions of interest (ROIs) were hand-outlined by a single investigator (NDP) trained to recognize the organs of interest by an experienced, fellowship trained radiologist (MG). Lengthy outlining sessions were avoided to prevent observer fatigue in processing the sizable data set. Measurement of the cross-sectional area of each organ of interest at each slice was achieved using the freehand ROI outlining tool incorporated into the PACS software. The left and right adrenal glands (LA and RA, respectively) were each traced as a single ROI, including the body and legs of the organ. An example of ROI outlining is shown in Figure 1. The same methods were used for volume measurement in contrast enhanced and unenhanced studies. On average, seven image slices were segmented per patient imaging study, for a total of over 160 adrenal volume measurements through the individual hand outlining of more than 1240 ROIs.

### Volume calculation

The cross-sectional area of each organ of interest on each individual CT image was obtained using the freehand ROI outlining tool of the PACS. The volume of each slice was then estimated as the product of the area of the ROI and the slice thickness. The total volume (V_total_) for each organ was computed as the sum of the volumes of all CT sections that included the organ:

$$V_{\mathrm{total}}\;=\;\sum \;{\mathrm{(A}}_{j}\;*\;\mathrm{t)},$$

where A_j_ is the area of the ROI for the organ of interest at the j^th^ slice and t is the slice thickness.

### Inter-observer variability

While a single observer performed the manual segmentation for volume assessment on all seven patients, variability between observers in outlining ROIs was studied by comparing the performances of two independent observers in outlining the organs of interest from CT scans of three of the patients. Each observer hand-outlined the organ of interest (LA and RA) from the same CT data sets of the same three patients without knowledge of each other’s measurements. In total, for the three image sets, each observer outlined 23 ROIs for the LA and 23 ROIs for the RA. The ROI areas were used to calculate the volume of the LA and RA, per each observer’s hand-outlining. The mean percent difference in calculated volume from each observer’s measurements ([observer 1 – observer 2]/mean) was used to assess inter-observer variability.

### Statistical analysis

The coefficient of variation (COV), defined as the standard deviation divided by the mean, was used to evaluate the precision in adrenal volume measurement across a series of CT scans. The coefficient of determination, also known as the square of the correlation coefficient (r^2^), was used to compare the variation in the measured volumes of paired organs over time. All statistics were calculated with standard spreadsheet software (Microsoft Excel, Microsoft Corporation, Redmond, Washington). Comparative analyses were made using the t-test with statistical significance assumed at *P* < 0.05 in all cases.

## Results

### Inter-observer variability

Inter-observer variability in outlining ROIs was evaluated by comparing volume measurements on CT volume data sets from three different patients by two independent observers. The LA and RA volumes were assessed by both observers. The mean percent differences ([observer 1 – observer 2]/mean) between observers for the three LA and three RA volume assessments was 24.7% and 19.1%, respectively.

### Adrenal gland volume assessment

The volumes of the LA and RA are plotted for three patients against the CT scan number in Figures 2a, 2b, and 2c. For comparison, the volume for a sphere of 10 mm in diameter is 0.52 cm^3^, a 20 mm diameter sphere has a volume of 4.2 cm^3^, and a 30 mm diameter sphere has a volume of 14.1 cm^3^. The mean volumes of the LA and RA were 5.9 ± 1.6 [standard deviation] cm^3^ and 4.5 ± 2.1 cm^3^, respectively, which correspond to equivalent spherical lesions with diameters of approximately 22.5 mm and 20.5 mm. The mean volumes of the LA and RA are provided in Table 2 for each patient. The smallest adrenal was 2.7 cm^3^ (Patient 2) and the largest was 8.0 cm^3^ (Patient 3).

**Figure 2a:**
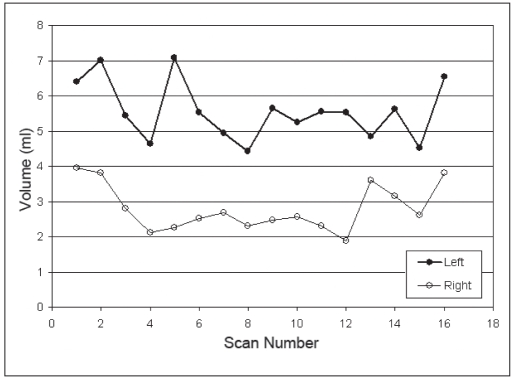
Adrenal volume over time. (Patient 1)

**Figure 2b:**
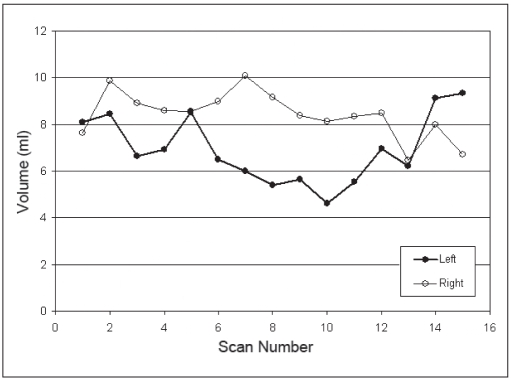
Adrenal volume over time. (Patient 2)

**Figure 2c:**
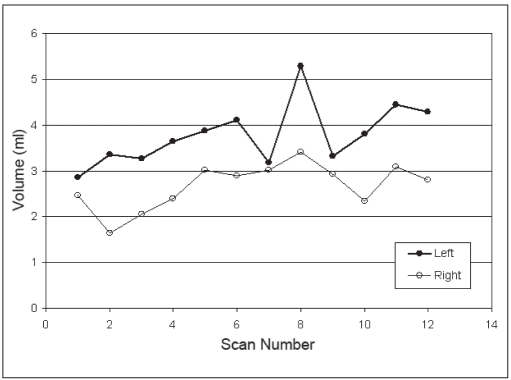
Adrenal volume over time. (Patient 3)

**Table 2:** Mean organ volumes: List of mean volumes, averaged over all CT scans for each patient

*Patient number*	*Mean organ volume (cm^3^)*	
	*Left adrenal*	*Right adrenal*
1	5.6	2.8
2	3.8	2.7
3	6.9	8.4
4	4.6	3.5
5	6.5	3.4
6	8.0	5.1
7	-	5.7
Mean	5.9	4.5
Std dev	1.6	2.1

**Figure 3:**
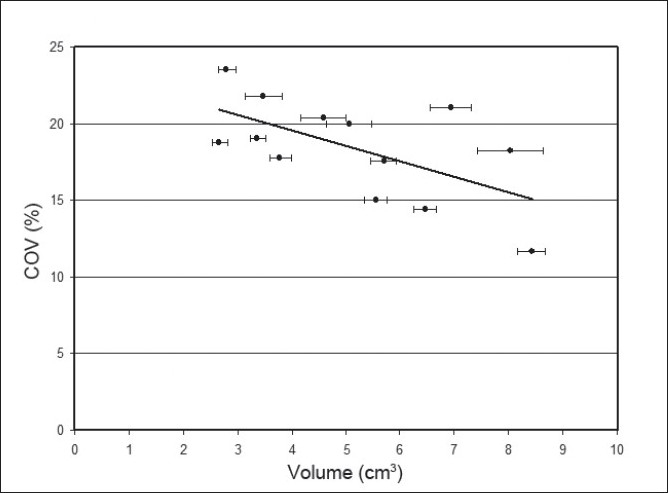
Coefficient of variation versus volume: Plotting the coefficient of variation as a function of adrenal gland volume shows an inverse relationship between the two variables. As volume increases, the coefficient of variation decreases, or equivalently, the precision increases. The coefficient of determination (r2) of the trend line is 0.367. The error bars represent the standard error of the adrenal gland volume measurement.

The COV of LA and RA volume measurement for each patient is tabulated in Table 3. The mean COV for the LA was 17.8%, and it was 18.9% for the RA. The COV was plotted as a function of adrenal gland volume for the 13 adrenal glands that were evaluated [Figure 3]. A negative correlation was found between the two quantities (r^2^=0.367) with COV decreasing as volume increased.

**Table 3:** Coefficient of variation for volume measurements

*Patient number*	*Coefficient of variation (COV)*	
	*Left adrenal*	*Right adrenal*
1	15.0%	23.5%
2	17.8%	18.8%
3	21.0%	11.7%
4	20.4%	21.8%
5	14.4%	19.1%
6	18.2%	20.0%
7	-	17.5%
Mean	17.8%	18.9%
Std dev	2.7%	3.8%

The coefficient of variation for each organ and patient is shown. The mean and standard deviation were calculated using all CT scans available for each patient

As an example, the 20 RA measurements (mean volume = 3.4 cm^3^, SD = 0.64 cm^3^) from Patient 5 were evaluated in a hypothetical exercise of the statistically detectable change in volume. Using the two-sided paired t-test, it was found that a 13.0% increase in true volume results in a statistical difference at *P*=0.05, and a 17.8% increase in true volume results in a statistical difference at *P*=0.01. Assuming isotropic growth of the tumor, these volume changes of 13.0% and 17.8% correspond to linear changes in the dimension of the tumor of 4.2% and 5.6%, respectively.

The coefficient of determination (r^2^) between LA and RA volume measurements over time, as represented by the series of CT scans performed, was calculated for all patients [Table 4]. Overall, little correlation between adrenal gland volume fluctuations was observed. The mean coefficient of determination for the population was 0.195 (SD = 0.195) and ranged from a minimum of 0.023 for Patient 4 to a maximum of 0.495 for Patient 6. Patient 7 does not have a coefficient of determination because the patient only had one adrenal gland.

**Table 4:** Correlation of adrenal gland volumes

Patient number	*Correlation coefficient for each organ pair*
	r^2^
1	0.197
2	0.362
3	0.046
4	0.023
5	0.050
6	0.495
7	-
Mean	0.195
Std dev	0.195

The coefficient of determination (r^2^) between left and right adrenal glands is shown. The typical correlation between the left and right adrenal glands was low (mean r^2^ = 0.195)

## Discussion

As seen in Figures 2a, 2b, and 2c, there are no obvious trends in the size of the adrenal organs over time – that is, the fluctuations in the RA volume are visibly uncorrelated to the fluctuations in the LA volume. Quantitatively, this is confirmed by the low mean coefficient of determination for the population (r^2^ = 0.195). If the correlation were high between LA and RA organ volumes, this would suggest that factors other than straightforward measurement precision may be causing temporal changes in measured volumes.

The RECIST criterion[19] defines “partial response” when there is a 30% decrease in a tumor’s maximum linear dimension. This work did not specifically address the fidelity in linear measurement; however, a change of 30% in all three linear dimensions of an object would result in a decrease in volume to 34.3% of its initial volume (0.343 = 0.70^3^). What the results of this work demonstrate is that for objects (e.g. tumors) in the size range of the adrenal glands (2.5 to 9 cm^3^), a subtle change in volume would be easily observable given the level of measurement precision documented here.

As described in the results, using the 20 RA measurements (mean volume = 3.4 cm^3^, SD = 0.64 cm^3^) from Patient 5 as an example, a statistically “true” change in volume would be detectable at a 13.0% increase in volume (*P*=0.05) and at a 17.8% increase in volume (*P*=0.01). Assuming isotropic growth of the tumor, these volume changes of 13.0% and 17.8% correspond to linear changes in the dimension of the tumor of 4.2% and 5.6%, respectively. A completely spherical tumor with a volume of 3.4 cm^3^ has a diameter of 12.8 mm. An abdominal CT scan produces pixel dimensions of about 0.68 mm, depending on the field of view (350 mm field of view / 512 pixels). A positioning error of even 1 pixel on the CT image therefore produces a 5.3% change in linear dimension, which is greater than the linear change (4.2%) associated with a spherical lesion growing by a statistically detectable amount (*P*=0.05), as described above. In other words, when using a linear measurement to measure the change in size of an object with volume close to that of the adrenal glands, small positioning errors, even by a single pixel, in that linear measurement are enough to incorrectly identify a statistically significant change in volume. As a result, this suggests that volume measurements will have more precision than linear measurements of tumor dimension, suffering less from such small errors in outlining fidelity. These findings propose that volume measurement is a more precise measure of volume change than linear measurement and thus support a paradigm shift toward the use of volumetric measurement as a preferred tool for tracking tumor growth.

The volume assessment reproducibility of approximately 18% found in this study is consistent with the findings of others who have studied volume assessment of tumors. Van de Langenberg reported 19.7% inter-observer variability in volume assessment of vestibular schwannomas imaged using MR.[20] Studies of other tumor types imaged with MR or CT have reported measurement variability between 13% and 28%.[11,12,21–23] Our study suggests that when imaging objects (e.g. tumors) with size comparable to that of the adrenal glands (2.5 to 9 cm^3^), up to an 18% variability in volume measurement can be expected that is otherwise not attributable to true change in the object’s size. The adrenal glands were used as surrogate tumors, assuming no actual volume change; however, reports on adrenal gland volume as a function of age are conflicting, with some studies reporting no significant volume change over time[14,15] and others reporting a small, but statistically significant, increase in volume with age. Meier demonstrated an increase in adrenal gland volume of approximately 0.03 ml per year between 20 and 80 years of age,[13] which corresponds to a 0.6% annual change in volume for an adrenal gland of average volume (5.2 cm^3^). For Patient 7, whose imaging studies spanned the longest period of time (5.25 years), this model would predict a 3% change in adrenal gland volume over that period, which is much smaller than the 18% volume measurement variability identified in our study.

This study also verifies that volume assessment precision improves with increasing object size, as one might expect [Figure 3]. This is likely due to the relative decrease in sampling of an object as object volume decreases, given a fixed CT slice thickness. With the use of thin slice acquisition and the addition of ideal segmentation software, better precision may be achievable.[24–27]

## Conclusion

In this study, a series of 92 CT scans performed serially in seven patients (13 glands) was used to evaluate CT-based volume measurement precision, using the left and right adrenal glands as surrogates for tumors. It was assumed that the adrenal glands did not have true volume changes over the measurement interval for each patient. It was found that the relative precision for human observers hand-outlining lesions, expressed as the coefficient of variation, averaged about 18% (17.8% for the LA, 18.9% for the RA) for organs with typical volume of 5.2 cm^3^ (range: 2.7 cm^3^ to 8.4 cm^3^). This result can be interpreted as the expected precision in repeated volumetric measurement of adrenal-sized objects, in a real-world clinical imaging setting. Since volume measurement is less susceptible to cursor positioning fidelity than linear measurement, it more precisely quantifies volume change. These results support the use of volumetric measurement rather than linear measurement in monitoring tumor growth, as in disease progression, or in monitoring response to therapy. This study also showed that precision improves with increasing object size. Clinically, volume measurement should be used to evaluate changes in tumor size, where 18% volume change serves as a threshold for adrenal-sized tumors, defining the measurement precision. Below the threshold, the observed volume change may be due to lack of measurement precision; above the threshold, the measurement may be a true volume change. Such a threshold would be smaller for increasing tumor size. Future studies exploring the dependence of volume measurement precision on scan protocol parameters, such as CT section thickness, the use of contrast agents, window/level settings, and segmentation algorithms, may build upon the findings of this study to better define the precision for specifically defined scan protocols.

## References

[1] Tsai CH, Lin CM, Hsieh CC, Hsu WH, Wang HW, Wang LS (2006). Tumor volume is a better prognostic factor than greatest tumor diameter in stage Ia non-small cell lung cancer. Thorac Cardiovasc Surg.

[2] Chen SW, Yang SN, Liang JA, Tsai MH, Shiau AC, Lin FJ (2006). Value of computed tomography-based tumor volume as a predictor of outcomes in hypopharyngeal cancer after treatment with definitive radiotherapy. Laryngoscope.

[3] Créhange G, Bosset M, Lorchel F, Buffet-Miny J, Dumas JL, Mercier M (2006). Tumor volume as outcome determinant in patients treated with chemoradiation for locally advanced esophageal cancer. Am J Clin Oncol.

[4] Cavalcanti MG, Santos DT, Perrella A, Vannier MW (2004). CT-based analysis of malignant tumor volume and localization. A preliminary study. Braz Oral Res.

[5] Ashamalla H, Guirgius A, Bieniek E, Rafla S, Evola A, Goswami G (2007). The impact of positron emission tomography/computed tomography in edge delineation of gross tumor volume for head and neck cancers. Int J Radiat Oncol Biol Phys.

[6] Villers A, Puech P, Mouton D, Leroy X, Ballereau C, Lemaitre L (2006). Dynamic contrast enhanced, pelvic phased array magnetic resonance imaging of localized prostate cancer for predicting tumor volume: correlation with radical prostatectomy findings. J Urol.

[7] Watanabe M, Kida M, Yamada Y, Saigenji K (2004). Measuring tumor volume with three-dimensional endoscopic ultrasonography: an experimental and clinical study (including video). Endoscopy.

[8] Ravenel JG, Leue WM, Nietert PJ, Miller JV, Taylor KK, Silvestri GA (2008). Pulmonary nodule volume: effects of reconstruction parameters on automated measurements--a phantom study. Radiology.

[9] Way TW, Chan HP, Goodsitt MM, Sahiner B, Hadjiiski LM, Zhou C (2008). Effect of CT scanning parameters on volumetric measurements of pulmonary nodules by 3D active contour segmentation: A phantom study. Phys Med Biol.

[10] Gietema HA, Schaefer-Prokop CM, Mali WP, Groenewegen G, Prokop M (2007). Pulmonary nodules: Interscan variability of semiautomated volume measurements with multisection CT-- influence of inspiration level, nodule size, and segmentation performance. Radiology.

[11] Vaidyanathan M, Clarke LP, Velthuizen RP, Phuphanich S, Bensaid AM, Hall LO, Bezdek JC (1995). Comparison of supervised MRI segmentation methods for tumor volume determination during therapy. Magn Reson Imaging.

[12] Gordon AR, Loevner LA, Shukla-Dave A, Redfern RO, Sonners AI, Kilger AM (2004). Intraobserver variability in the MR determination of tumor volume in squamous cell carcinoma of the pharynx. AJNR Am J Neuroradiol.

[13] Meier JM, Alavi A, Iruvuri S, Alzeair S, Parker R, Houseni M (2007). Assessment of age-related changes in abdominal organ structure and function with computed tomography and positron emission tomography. Semin Nucl Med.

[14] Nemeroff CB, Krishnan KR, Reed D, Leder R, Beam C, Dunnick NR (1992). Adrenal gland enlargement in major depression. A computed tomographic study. Arch Gen Psychiatry.

[15] Bertolini G, Furlanello T, Drigo M, Caldin M (2008). Computed tomographic adrenal gland quantification in canine adrenocorticotroph hormone-dependent hyperadrenocorticism. Vet Radiol Ultrasound.

[16] Geraghty EM, Boone JM, McGahan JP, Jain K (2004). Normal organ volume assessment from abdominal CT. Abdom Imaging.

[17] Yuan Y, Giger ML, Li H, Suzuki K, Sennett C (2007). A dual-stage method for lesion segmentation on digital mammograms. Med Phys.

[18] Zhou X, Han M, Hara T, Fujita H, Sugisaki K, Chen H (2008). Automated segmentation of mammary gland regions in non-contrast X-ray CT images. Comput Med Imaging Graph.

[19] Therasse P, Arbuck SG, Eisenhauer EA, Wanders J, Kaplan RS, Rubinstein L (2000). New guidelines to evaluate the response to treatment in solid tumors. European Organization for Research and Treatment of Cancer, National Cancer Institute of the United States, National Cancer Institute of Canada. J Natl Cancer Inst.

[20] van de Langenberg R, de Bondt BJ, Nelemans PJ, Baumert BG, Stokroos RJ (2009). Follow-up assessment of vestibular schwannomas: volume quantification versus two-dimensional measurements. Neuroradiology.

[21] Bauknecht HC, Romano VC, Rogalla P, Klingebiel R, Wolf C, Bornemann L (2009). Intra- and Interobserver Variability of Linear and Volumetric Measurements of Brain Metastases Using Contrast-Enhanced Magnetic Resonance Imaging. Invest Radiol.

[22] Goodman LR, Gulsun M, Washington L, Nagy PG, Piacsek KL (2006). Inherent variability of CT lung nodule measurements *in vivo* using semiautomated volumetric measurements. AJR Am J Roentgenol.

[23] Mazzara GP, Velthuizen RP, Pearlman JL, Greenberg HM, Wagner H (2004). Brain tumor target volume determination for radiation treatment planning through automated MRI segmentation. Int J Radiat Oncol Biol Phys.

[24] Alderliesten T, Schlief A, Peterse J, Loo C, Teertstra H, Muller S (2007). Validation of semiautomatic measurement of the extent of breast tumors using contrast-enhanced magnetic resonance imaging. Invest Radiol.

[25] Clark MC, Hall LO, Goldgof DB, Velthuizen R, Murtagh FR, Silbiger MS (1998). Automatic tumor segmentation using knowledge-based techniques. IEEE Trans Med Imaging.

[26] Peck DJ, Windham JP, Emery LL, Soltanian-Zadeh H, Hearshen DO, Mikkelsen T (1996). Cerebral tumor volume calculations using planimetric and eigenimage analysis. Med Phys.

[27] Yan J, Zhao B, Wang L, Zelenetz A, Schwartz LH (2006). Marker-controlled watershed for lymphoma segmentation in sequential CT images. Med Phys.

